# High doses of phytase on growth performance, bone mineralization, diet utilization, and plasmatic *myo*-inositol of turkey poults

**DOI:** 10.1016/j.psj.2021.101050

**Published:** 2021-02-16

**Authors:** Lucas S. Bassi, Levy V. Teixeira, Rafael F. Sens, Leopoldo Almeida, Vitor A.B. Zavelinski, Alex Maiorka

**Affiliations:** ∗ Department of Animal Science, Federal University of Paraná, Curitiba, Brazil 80035-050; † DSM Nutritional Products, São Paulo, Brazil 03178-200

**Keywords:** digestibility, *myo*-inositol, P, phytase, turkey

## Abstract

An experiment was conducted to evaluate the growth performance, bone mineral composition, diet utilization, and plasmatic concentration of *myo*-inositol (**MYO**) in turkeys fed different phytase doses from 1 to 28 d. A total of three hundred and twenty 1-day-old turkeys were distributed in a completely randomized design with 4 treatments and 8 replicates of 10 birds each. Treatments included a basal diet without phytase; reduced diet (reduced −0.15% available P and −0.18% Ca) without phytase; reduced diet + 2,000 units of phytase (**FYT**)/kg; and reduced diet + 4,000 FYT/kg. From day 26 to 28, partial excreta collection was conducted, and on day 28, 7 birds per replicate were euthanized for collection of ileal content and left tibia bones were removed from 2 of the same euthanized birds. Feed, excreta, and ileal digesta samples were analyzed to determine nutrient digestibility and metabolizability, ileal digestible energy, and AME. Tibia bones were analyzed for ash, Ca, and P content, and calculation of Seedor index. On day 28, blood samples were collected from 2 turkeys per replicate to analyze plasmatic MYO concentration. Feed conversion ratio was not affected, but phytase supplementation resulted in higher feed intake and body weight gain compared to turkeys fed the reduced diet (*P* < 0.05), and both doses were similar to the basal diet. Increasing the phytase dose had a linear effect (*P* < 0.05) on ileal digestibility of P and metabolizability of DM, CP, Ca, and Na, and also on AME. P content in the tibia bone increased linearly (*P* < 0.05) with phytase supplementation, and the same linear increase (*P* < 0.05) was observed for plasmatic MYO. In conclusion, the supplementation of turkey poult's diets with high levels of phytase up to 4,000 FYT/kg improves diet utilization by increasing P digestibility and dietary metabolizability, leading to higher P content in the bone and enhancing MYO provision and absorption.

## Introduction

Diets for turkeys, similar to broiler chicken diets, are primarily composed of plant-based ingredients, such as corn and soybean meal. In these ingredients, most of the P is unavailable, as it is covalently bound to inositol, forming the anti-nutritional factor known as phytate (*myo*-inositol [**MYO**] hexakiphosphate). The anti-nutritional effect of phytate also extends to the chelation of dietary minerals and interaction with dietary amino acids, negatively affecting their solubility and reducing digestion and absorption (Cowieson et al., 2009). The endogenous production of phytase and phosphatase enzymes in the tract of monogastric animals is insufficient to properly hydrolyze the phytate molecule; so supplementation of exogenous phytase in poultry diets has become a routine practice.

Phytase is known to improve growth performance, nutrient digestibility, and bone mineralization in turkeys (Pirgozliev et al., 2007; Kozlowski et al., 2010; Wealleans et al., 2016). Recent studies have shown that phytase doses higher than 1,500 units of phytase (**FYT**)/kg may provide extra-phosphoric effects or additional benefits beyond the increase in P solubility in diets for broilers (Cowieson et al., 2017; Dersjant-Li and Kwakernaak, 2019; Walk and Rama Rao, 2020) and turkeys (Pirgozliev et al., 2011). These investigations describe an increase in phytate dephosphorylation with high inclusion of phytase, marked by the reduction of phytate esters and their elimination from the gastrointestinal tract (**GIT**). In turn, dietary solubility in the tract is overall increased, which boosts nutrient utilization and growth performance.

*myo*-Inositol is the final product of the complete dephosphorylation of phytate. According to Gonzalez-Uarquin et al. (2020), MYO holds an important role in metabolic and regulatory processes within the animal organism, for example acting as an insulin mimetic and modulating glucose homeostasis; associations with lipid metabolism and bone mineral absorption; antioxidant effects; and osmotic balance regulation in specific tissues. Studies with broiler chickens have shown the effect of high levels of phytase on increasing concentrations of MYO in the digesta as a consequence of higher phytate degradation rates, and because MYO is linked to several metabolic functions, improvements in growth performance can be achieved (Cowieson et al., 2013, 2015; Sommerfeld et al., 2018).

Studies involving supplementation of high doses of phytate in diets for turkeys are scarce when compared to broiler chickens, even though the capacity to utilize dietary P is different between poultry species. Turkeys have a lower rate of P utilization when compared to broiler chickens, ducks, and quails, possibly because of differences in the passage rate and pH along the tract, lower activity of endogenous phytase, and especially because of an inferior capacity to hydrolyze phytate (Rodehutscord and Dieckmann, 2005).

Thus, the objective of this study was to assess the effects of high doses of phytase on growth performance, nutrient digestibility and metabolizability, bone mineralization, and MYO levels in the plasma of turkeys.

## Materials and methods

All experimental procedures were approved by the Animal Use Ethics Committee of Federal University of Paraná.

### Animals, Facilities, and Experimental Design

A total of 320 male turkeys (Nicholas Select, Aviagen America Latina Ltda, Rio Claro, SP, Brazil) were allocated in metabolic battery cages from 1 to 28 d of age. Each battery consisted of 4 floors with 2 cages per floor and 10 turkeys per cage. Each cage measured 0.98 m in length × 0.90 m in width × 0.50 m in height and was equipped with gutter feeders and nipple drinkers. During the first 3 d, incandescent light was provided for 24 h, after which a lighting program of 6 h of darkness per day was applied. Room temperature was set to 30°C on day 1 and weekly reduced to 23°C on day 28.

A completely randomized design with 4 treatments and 8 replicates of 10 turkeys each was evaluated. The 4 dietary treatments were a basal diet without phytase; reduced diet without phytase, with reduction of 0.18% Ca and 0.15% available P; reduced diet + 2,000 FYT/kg; reduced diet + 4,000 FYT/kg. The phytase utilized in the experiment was HiPhos GT (Ronozyme HiPhos Granulated Thermostable, DSM, Kaiseraugst, Switzerland), which is a 6-phytase that originated from *Citrobacter braakii* and is expressed in *Aspergillus oryzae*, with minimum activity of 20,000 FYT/g of the product. One phytase unit (FYT, **FTU**) was defined as the quantity of enzyme which liberates 1 μmol of inorganic phosphate per minute from 5.0 μmol/L Na phytate at pH 5.5 and 37°C (Engelen et al., 1994). Experimental diets were fed in mash form and based on corn and soybean meal (Table 1). Feed and water were offered ad libitum during the whole experimental period. The activity of phytase in the supplemented diets (Table 2) was measured at Biopract GmbH (Berlin, Germany) with the method PHY-101/05E, in accordance with the method ISO30024:2009 by the International Organization for Standardization (ISO, 2009).

**Table 1:** Ingredients and composition of the experimental diets.

Ingredients	Basal diet	RD	RD + 2000 FYT	RD + 4000 FYT
Corn (%)	46.838	48.317	48.307	48.297
Soybean meal (%)	41.400	41.200	41.200	41.200
Corn gluten meal (%)	3.000	3.000	3.000	3.000
Soybean oil (%)	0.700	0.200	0.200	0.200
Dicalcium phosphate (%)	3.570	2.770	2.770	2.770
Limestone (%)	1.300	1.320	1.320	1.320
Sodium chloride (%)	0.470	0.470	0.470	0.470
Celite^1^ (%)	1.000	1.000	1.000	1.000
DL-methionine (%)	0.420	0.420	0.420	0.420
L-lysine HCl (%)	0.517	0.520	0.520	0.520
L-threonine (%)	0.116	0.114	0.114	0.114
L-valine (%)	0.155	0.154	0.154	0.154
L-arginine (%)	0.138	0.139	0.139	0.139
Choline chloride (%)	0.080	0.080	0.080	0.080
Vitamin premix^2^ (%)	0.150	0.150	0.150	0.150
Mineral premix^3^ (%)	0.080	0.080	0.080	0.080
Protease^4^ (%)	0.020	0.020	0.020	0.020
Amylase^5^ (%)	0.013	0.013	0.013	0.013
25(OH)D_3_ premix^6^ (%)	0.033	0.033	0.033	0.033
Phytase^7^	0.000	0.000	0.010	0.020
Calculated chemical composition				
Metabolizable energy (kcal/kg)	2,901	2,901	2,901	2,901
CP (%)	27.623	27.642	27.642	27.642
Ether extract (%)	3.487	3.044	3.044	3.044
Ca (%)	1.519	1.338	1.338	1.338
Available P (%)	0.800	0.650	0.650	0.650
Na (%)	0.200	0.200	0.200	0.200
Chlorine (%)	0.345	0.346	0.346	0.346
Digestible lysine (%)	1.700	1.700	1.700	1.700
Digestible methionine (%)	0.797	0.797	0.797	0.797
Digestible methionine + cysteine (%)	1.186	1.187	1.187	1.187
Digestible threonine (%)	1.054	1.053	1.053	1.053
Digestible tryptophan (%)	0.281	0.280	0.280	0.280
Analyzed chemical composition				
CP (%)	27.66	27.09	27.91	27.79
Ca (%)	2.03	1.80	1.78	1.77
Total P (%)	1.20	1.05	1.05	1.07
Na (%)	0.25	0.26	0.28	0.27

Abbreviations: FYT, units of phytase; RD, reduced diet.

1 Celite insoluble marker (Celite 400, Celite Corp., Lompoc, CA).

2 Provided per kg of premix: vitamin A, 11,000,000 UI; vitamin D3, 4,000,000 UI; vitamin E, 55,000 UI; vitamin K3, 3 g; vitamin B1, 2.3 g; vitamin B2, 7 g; pantothenic acid, 12 g; vitamin B6, 4 g; vitamin B12, 25 mg; nicotinic acid, 60 g; folic acid, 2 g; biotin, 250 mg; selenium, 300 mg.

3 Provided per kg of premix: copper, 20 g; iron, 100 g; iodine, 2 g; manganese, 130 g; zinc, 130 g.

4 ProAct (CT) (Ronozyme ProAct, DSM, Kaiseraugst, Switzerland) at 15,000 PROT/kg.

5 HiStarch (CT) (Ronozyme HiStarch, DSM) at 80 KNU/kg.

6 Hy-D Premix (Hy-D, DSM) at 91 mg of 25(OH)D_3_/ton of feed.

7 HiPhos (GT) (Ronozyme HiPhos, DSM) with 20,000 FYT/g of product.

**Table 2:** Expected and analyzed phytase activity recovered in feed samples.

Treatment	Phytase^2^, FYT^3^/kg	
	Declared	Analyzed
Basal diet	0	<LOD^4^
RD^1^	0	<LOD
RD + 2,000 FYT	2,000	2,386
RD + 4,000 FYT	4,000	4,014

Abbreviation: RD, reduced diet.

1 Reduction of 0.18% in Ca and 0.15% in available P levels.

2 Enzyme activity is expressed as the quantity of product added in the feed.

3 FYT = phytase units per kg of feed.

4 LOD = limit of detection.

### Growth Performance

Growth performance variables were recorded from 1 to 28 d by weighting all the turkeys and feed leftovers at day 1 and again at day 28 to calculate feed intake (**FI**), body weight gain (**BWG**), and feed conversion ratio (**FCR**) corrected to the weight of dead turkeys.

### Digestibility and Metabolizability

Partial excreta collection was conducted from day 26 to 28, with one collection per day at an identical timing on each day. Approximately 20% of the excreta was randomly collected from each cage. Samples were placed in identified plastic bags, avoiding contamination with feathers and feed, and stored in a freezer at −18°C. At day 28, 7 turkeys per replicate were randomly selected and euthanized to collect the ileal content. Turkeys were eviscerated, and an ileal fraction was separated for content removal, defined as 4 cm below Meckel's diverticulum and 4 cm above the ileum-cecum-colon junction. Ileal content samples from all 7 turkeys were pooled, placed in identified plastic containers, and frozen at −18°C.

Excreta and ileal digesta samples were subsequently thawed to room temperature and dried in a force-ventilation oven at 55°C for 72 h. Feed, excreta, and ileal samples were then ground to a particle size of 0.5 mm. DM was determined by oven drying at 105°C for 12 h, and CP (method 954.01), Ca (method 927.02), and P (method 965.17) contents were analyzed according to the Association of Official Analytical Chemists (AOAC, 1995). Na (method 969.23) content was also analyzed in the feed and excreta samples, according to AOAC (1995). Gross energy of the samples was determined in a calorimetric bomb (Ika Werke C 2000 Control Oxygen Bomb Calorimeter, Ika-Werke GmbH & Co., Staufen, Germany). Acid-insoluble ash (**AIA**) was used as an insoluble marker compound in the digestibility calculations, and AIA content in the samples was determined according to the methodology of Scott and Boldaji (1997).

Based on the results, the coefficients of apparent ileal digestibility (**CAID**) and apparent metabolizability (**CAM**) were calculated according to the following formula:

$$\text{CAM or CAID}\left(\%\right)=\frac{\left(\text{Nutrient in the diet}\right)-\left(\text{Nutrient in the excreta or ileal digesta}\times \text{IF}\right)}{\text{Nutrient in the diet}}$$

where indigestibility factor, IF, is the ratio between AIA content in the diet and AIA in the excreta or ileal digesta. Ileal digestible energy (**IDE**) and AME were calculated according to the following formula:

AME or ADE(kcal/kg DM)=GE in the diet−(GE in the excreta or ileal digesta×IF)

### Bone Mineral Composition

Two of the turkeys euthanized at day 28 for ileal content collection had their left tibia bones manually removed. Tibial bones were cleaned with ether to remove any remnants of fat and muscle, and oven-dried at 105°C for 12 h. Bone length and weight measurements were then taken by using a digital caliper and a digital scale to the nearest 0.0001 g, and the Seedor index (**SI**) was calculated by dividing the bone weight by its length, as an indicator of bone mineral density (Seedor, 1993). Dried bones were ashed in a muffle furnace at 600°C for 3 h, and ash (method 942.05), Ca (method 927.02), and P (method 965.17) contents were analyzed according to AOAC (1995).

### Circulating MYO

At 28 d, 2 birds per replicate were randomly chosen to collect (3–5 mL) blood samples. The blood was collected by puncture of the wing vein and held in tubes with heparin (Vacutainer Plus, Becton Dickinson, Franklin Lakes, NJ). Immediately after blood collection, the tubes were centrifuged at 1,008 rcf for 10 min, in order to separate the plasma from blood. After centrifugation, the obtained plasma was stored in graduated microtubes at −20°C until analysis. Concentration of MYO in the plasma was determined by mass spectrometry using an UPLC system (ACQUITY UPLC System, Waters, Milford, MA) according to the method described by Leung et al. (2011).

### Statistical Analysis

The residue normality of the data was determined by Shapiro-Wilk test and after a normal distribution was detected, all data were submitted to ANOVA using the linear model of ExpDes package (Ferreira et al., 2013) on R program (R Foundation for Statistical Computing, Vienna, Austria). Prior to the ANOVA, an outlier removal method was applied by viewing the data in a box plot and then removing data points that were located outside the whiskers of the box plot. When significant, means were compared by Tukey test at 5% probability. Linear and quadratic analyses of regression were conducted between the reduced diet treatment and the 2 levels of phytase supplementation.

## Results

### Growth Performance

The growth performance results (Table 3) indicate that phytase-supplemented diets increased FI and BWG (*P* < 0.05) by 4.05 and 9.61%, respectively, comparing the inclusion of 4,000 FYT/kg and the reduced diet. FI and BWG were statistically similar between the 2,000 and 4,000 FYT dose and both supplementation levels were similar to the basal diet. FCR was not influenced by the dietary treatments. The mortality rate throughout the experimental period was 6.56%.

**Table 3:** FI, WG, and FCR of turkeys fed different doses of phytase from 1 to 28 d.

Treatment	FI (g)	WG (g)	FCR (g/g)
Basal diet	1,226.01^a^	815.75^a^	1.510
RD^1^	1,167.92^b^	738.60^b^	1.582
RD + 2,000 FYT^2^	1,231.20^a^	810.87^a^	1.518
RD + 4,000 FYT	1,219.73^a^	806.48^a^	1.516
SEM	17.12	15.45	0.014
*P*-value	0.003	0.042	0.776
Linear *P*-value	0.186	0.230	0.242
Quadratic *P*-value	0.399	0.269	0.368

^a,b^Means within a column lacking a common superscript differ (*P* < 0.05).

Abbreviations: FCR, feed conversion ratio; FI, feed intake; RD, reduced diet; WG, weight gain.

1 Reduction of 0.18% in Ca and 0.15% in available P levels.

2 FYT = phytase units per kg of feed.

### Digestibility and Metabolizability

The phytase-supplemented diets promoted higher CAID of P (*P* < 0.001) in comparison to the control groups (Table 4). Increasing the phytase doses had a linear effect (*P*-linear < 0.05) on ileal P digestibility. However, no differences were observed between the supplemented diets and the basal or reduced diets for CAID of DM, CP, and Ca, nor IDE, although a crescent linear trend (*P*-linear < 0.1) was observed for the CAID of CP with increasing doses of phytase.

**Table 4:** Coefficient of apparent digestibility of DM, CP, Ca, and P, and IDE of 28-day-old turkeys fed different phytase doses.

Treatment	DM (%)	CP (%)	Ca (%)	P (%)	IDE (kcal)
Basal diet	74.27	89.38	62.06	69.78^b^	3,312
RD^1^	73.16	89.56	64.20	70.83^b^	3,222
RD + 2,000 FYT^2^	73.20	88.70	65.14	73.63^a,b^	3,239
RD + 4,000 FYT	72.65	90.26	63.81	75.60^a^	3,334
SEM	0.77	0.72	1.21	1.05	41.66
*P*-value	0.539	0.099	0.148	<0.001	0.492
Linear *P*-value	0.281	0.095	0.234	0.020^3^	0.151
Quadratic *P*-value	0.866	0.143	0.685	0.795	0.622

^a,b^Means within a column lacking a common superscript differ (*P* < 0.05).

Abbreviations: IDE, ileal digestible energy; RD, reduced diet.

1 Reduction of 0.18% in Ca and 0.15% in available P levels.

2 FYT = phytase units per kg of feed.

3 y = 0.0012x + 70.968 (*R*^2^ = 0.99).

As for the metabolizability results (Table 5), a significant effect was obtained for CAM of all analyzed variables (*P* < 0.01), including AME (*P* < 0.01). The inclusion of a higher phytase dose (4,000 FYT) substantially increased CAM of DM, CP, Ca, P, and Na, and AME, when compared to the zero-inclusion diets or the 2,000 FYT dose. A linear effect (*P*-linear < 0.001) was observed with increasing phytase doses for CAM variables and AME.

**Table 5:** Coefficient of apparent metabolizability of DM, CP, Ca, P, and Na, and AME (kcal/kg) of 28-day-old turkeys fed different phytase doses.

Treatment	DM (%)	CP (%)	Ca (%)	P (%)	Na (%)	AME (kcal)
Basal diet	68.18^b,c^	56.34^c^	67.10^c^	58.01^c^	65.08^b^	3,218^b^
RD^1^	65.36^c^	55.47^c^	69.42^c^	59.54^c^	64.71^b^	3,104^c^
RD + 2,000 FYT^2^	67.73^c^	61.99^b^	69.99^b,c^	61.50^b,c^	65.70^b^	3,256^b^
RD + 4,000 FYT	71.74^a^	68.88^a^	75.33^a^	73.21^a^	72.63^a^	3,486^a^
SEM	0.59	1.19	0.79	1.23	0.77	28.66
*P*-value	<0.001	<0.001	<0.001	<0.001	<0.001	<0.001
Linear *P*-value	<0.001^3^	<0.001^4^	0.003^5^	<0.001^6^	0.001^7^	<0.001^8^
Quadratic *P*-value	0.485	0.929	0.134	0.251	0.469	0.353

^a–c^Means within a column lacking a common superscript differ (*P* < 0.05).

Abbreviation: RD, reduced diet.

1 Reduction of 0.18% in Ca and 0.15% in available P levels.

2 FYT = phytase units per kg of feed.

3 y = 0.0016x + 65.087 (*R*^2^ = 0.9784).

4 y = 0.0034x + 55.408 (*R*^2^ = 0.9997).

5 y = 0.0015x + 68.625 (*R*^2^ = 0.8216).

6 y = 0.0034x + 57.915 (*R*^2^ = 0.855).

7 y = 0.002x + 63.72 (*R*^2^ = 0.8421).

8 y = 0.0955x + 3,092.7 (*R*^2^ = 0.9856).

### Bone Mineral Composition

Turkeys fed diets containing 4,000 FYT had a greater P content (*P* < 0.05) in tibiae bones when compared to those fed the reduced diet, whereas supplementation of 2000 FYT/kg provided the same P content as the basal and reduced diet groups (Table 6). The effect of increasing phytase doses on P content in the bone was linear (*P*-linear < 0.05). No difference was found between treatments for ash and Ca content or SI.

**Table 6:** Ash, Ca, and P percentage, and SI in tibia bone of 28-day-old turkeys fed different phytase doses.

Treatment	Ash (%)	Ca (%)	P (%)	SI (mm/mg)
Basal diet	48.67	18.03	8.71^a,b^	33.88
RD^1^	48.78	17.76	8.36^b^	33.37
RD + 2,000 FYT^2^	48.49	17.81	8.60^a,b^	34.17
RD + 4,000 FYT	49.15	18.42	8.96^a^	32.69
SEM	0.54	0.30	0.15	0.07
*P*-value	0.988	0.599	0.031	0.828
Linear *P*-value	0.835	0.089	0.005^3^	0.842
Quadratic *P*-value	0.519	0.417	0.642	0.329

^a,b^Means within a column lacking a common superscript differ (*P* < 0.05).

Abbreviations: RD, reduced diet; SI, Seedor index.

1 Reduction of 0.18% in Ca and 0.15% in available P levels.

2 FYT = phytase units per kg of feed.

3 y = 0.0002x + 8.34 (*R*^2^ = 0.9868).

### Circulating MYO

There was a significant difference between treatments for MYO levels in the plasma of turkeys (*P* < 0.05; Table 7), as the phytase-supplemented diets resulted in a higher concentration of plasmatic MYO in comparison to the basal and reduced diets. The effect of increasing phytase doses on MYO was linear (*P*-linear < 0.05).

**Table 7:** Concentration of MYO in the blood plasma of 28-day-old turkeys fed different phytase doses.

Treatment	MYO (μmol/L)
Basal diet	167.18^c^
RD^1^	170.50^b,c^
RD + 2,000 FYT^2^	211.82^a^
RD + 4,000 FYT	239.09^a^
SEM	7.69
*P*-value	<0.001
Linear *P*-value	<0.001^3^
Quadratic *P*-value	0.496

^a–c^Means within a column lacking a common superscript differ (*P* < 0.05).

Abbreviations: MYO, *myo*-inositol; RD, reduced diet.

1 Reduction of 0.18% in Ca and 0.15% in available P levels.

2 FYT = phytase units per kg of feed.

3 y = 0.0172x + 172.76 (*R*^2^ = 0.9861).

## Discussion

The reduction of Ca and P levels in the reduced diet expectedly restricted turkey growth, but supplementation of 4,000 FYT/kg to the diet increased FI and BWG, although FCR was not affected. Wealleans et al. (2016) reported an even more significant increase in FI and BWG of 21-day-old turkeys supplemented with phytase, as BWG increased by 20% with inclusion of 1,035 FTU/kg. The authors did not observe any effect of phytase supplementation on FCR. Even though the first weeks are critical for the development and maturation of the GIT, the reduced Ca and P levels did not impact on FCR. On the other hand, Kozlowski et al. (2010) observed that the inclusion of 500 and 1,000 FTU/kg in low Ca and P diets for turkeys improved FCR when compared to non-supplemented basal diets, as a reflection of the higher P supply to the birds. However, turkeys were reared up to 16 wk of age, and besides older turkeys having a more mature GIT, providing lower Ca and P dietary levels probably had a more evident long-term impact, thus enhancing phytase effects on FCR.

It was possible to observe that increasing doses of phytase on a low Ca and P diet had a linear effect on CAID and CMA of P. Positive results were also found by other studies regarding phytase for turkeys and its effects on P availability, although most of them used doses no higher than 1,000 FTU/kg (Applegate et al., 2003; Kozlowski et al., 2010; Wealleans et al., 2016). Choct (2006) explained that an inclusion of 500 FTU per kg of feed may liberate 0.1% of the available P bound to phytate, and in this current study, the inclusion of 4000 FYT/kg increased digestibility and metabolizability of P by 6.7 and 14.6%, respectively, compared to non-supplemented diets, which means more phytate P was successfully released and absorbed by the turkeys' digestive tract. The increase in P retention is substantially important, as excessive P excretion is allegedly one of the main hazards to soil and water quality, especially in areas with a large scale of poultry farming (Toor et al., 2005).

Literature describes the benefits of high levels of phytase supplementation on nutrient utilization beyond P. The extra-phosphoric effects of higher phytase doses are supported by an increase of solubility and reduced excretion of other minerals, amino acids, Na, as well as higher digestible and metabolizable energy, which is mainly related to a higher degree of phytate dephosphorylation and suppression of its anti-nutritional effects (Cowieson et al., 2011). These effects have been observed in diets for broilers (Walk et al., 2013; Cowieson et al., 2017; Dersjant-li and Kwakernaak, 2019; Walk and Rama Rao, 2020) and turkeys (Pirgozliev et al., 2007, 2011), and also in the current study, as increasing the phytase dose resulted in a linear trend for greater CAID of CP.

Even with phytase supplementation, the lack of difference in CAID of DM, Ca, or IDE could be linked to an immature GIT. In the first weeks post-hatch, the GIT of turkeys still undergoes slow development (Applegate et al., 2005), and the absorptive activity and enzyme secretion in the intestine of young poults are suboptimal (Sklan, 2001). However, phytase increased nutrient metabolizability and AME. As reported by Borda-Molina et al. (2019), P supplementation seems to bolster the population of beneficial microorganisms in the ceca of broilers, so the improvement on nutrient metabolizability could be related to better development of the microbiota in the distal gut of turkeys, possibly caused by a higher flow of P coming from the proximal gut. Presumably, phytase affected the microbial population of turkeys through greater nutrient provision. A recent study by González-Ortiz et al. (2020) evaluated the effects of phytase (up to 3,000 FTU/kg) and xylanase (100 g/ton) on ceca microbiota of 28-day-old broilers and turkeys. The authors observed that xylanase had positive effects on microbial populations of both species through the provision of oligosaccharides, but the microbiome of turkeys was unaffected by phytase, contrary to that observed in broilers. This might be again related to differences in gut maturation and also in microbiome colonization between species.

The excretion of Na was decreased by phytase supplementation, as observed in the CMA results. Phytate is known to increase Na excretion because of a greater transfer of Na bicarbonate to the gut lumen, in order to mitigate the phytate-induced excess of HCl (Allen and Flemstron, 2005). Phytase seems to counteract this effect, as seen in broilers' diets (Cowieson et al., 2004; Ravindran et al., 2006), and also in the results of this study, where high doses of phytase successfully improved CMA of Na, in comparison to the non-supplemented diets.

Supplementation of phytase in poultry diets improves bone mineralization by increasing the availability of dietary Ca and P and their absorption rate to the bone (Han et al., 2009; Farhadi et al., 2017). As observed in this study, the P percentage in the tibia bone of turkeys fed the low Ca and P diet increased with phytase inclusion. These results are consistent with those of Kozlowski et al. (2010), who reported an increase in tibia P content of turkeys fed up to 1,000 FTU/kg and Applegate et al. (2003), who observed a linear increase of tibia ash and toe ash of turkeys fed up to 500 U/kg. In the current study, a higher dose of the enzyme (4000 FYT) was more effective in sparing phytate P to the turkeys when comparing the P content in tibia provided by 2,000 FYT and the basal diet.

Treatments, however, neither affected ash and Ca content in bones nor SI. These results are contrary to those of Walk and Rama Rao (2020), who observed that phytase doses higher than 2,000 FTU resulted in higher Ca and ash content in the tibia of broiler chickens. Rezvani et al. (2009) tested the effects of 2 different phytase preparations on P utilization by turkeys, and observed that Ca and P content in the tibia was not affected, whereas ash concentration was affected only by one of the phytases, as enzymes from different sources have different characteristics such as thermostability and optimal pH of activity. The lack of variation in ash and Ca content in the bone, as well as the lack of difference in bone mineral composition between turkeys fed the basal and reduced diet may suggest that Ca and P levels were not sufficiently low to impair bone mineralization.

The concentration of MYO in turkey plasma was linearly increased by higher doses of phytase, most likely due to a higher dephosphorylation rate of phosphate isomers and the consequent release of inositol in the gut. These results are in line with other findings reporting higher MYO plasma levels when using phytase-supplemented diets for broilers (Cowieson et al., 2013, 2015; Sommerfeld et al., 2018). MYO is a product of the complete degradation of inositol phosphates (**IP**) and its role in metabolic processes involves insulin-mimetic effects, regulation of glucose transportation and lipid metabolism, osmotic balance in specific tissues such as brain and renal tissues, absorption of minerals, and antioxidant effects, among others (Gonzalez-Uarquin et al., 2020). The supplementation of broiler diets with MYO has positive effects on growth performance (Cowieson et al., 2013; Farhadi et al., 2017; Pirgozliev et al., 2017), although it seems to antagonistically react with phytase. Pirgozliev et al. (2017) observed that simultaneously supplementing broiler diets with MYO had a diminishing effect on P digestibility, and Cowieson et al. (2013) found a negative interaction between MYO and phytase for insulin blood levels, possibly mediated via competitive mechanisms. Nonetheless, MYO provision and concentration in turkey's plasma were enhanced by the high dose of phytase. In addition, plasma MYO was higher in turkeys fed the reduced diet compared to the basal diet. As higher Ca and P dietary levels decrease the efficacy of phytases and phosphatases, there is a consequent reduction of MYO concentration in the digesta (Gonzalez-Uarquin et al., 2020); thus, lowering these levels in the reduced diet increased plasma MYO concentration, which was further increased by phytase addition.

Turkeys' response to phytase supplementation differed from that of broilers, as dephosphorylation of IP isomers, such as IP_5_ and IP_4_, as well as plant P digestibility, is lower in turkeys. The results of a comparative study between broilers and turkeys conducted by Ingelmann et al. (2019) indicated that the pre-cecal digestibility of P in diets without phytase inclusion ranged from 51 to 60% for broilers and 22 to 28% for turkeys, and phytate degradation ranged from 64 to 70% for broilers and 6 to 15% for turkeys. Upon adding phytase, the increase in P digestibility was higher for turkeys than broilers, although the difference was small (17 × 15%, respectively). This indicates a lower capacity of turkeys to hydrolyze phytate in the GIT, which can be linked to species-related factors such as endogenous P loss and passage rate (Rodehutscord and Dieckmann, 2005; Ingelmann et al., 2019) or even differences in tract development and maturity (Adebiyi and Olukosi, 2015; Zeller et al., 2016). It must be then emphasized that the supplementation of turkey diets with phytase should be considered independently of broiler diets, as optimal responses to the enzyme may differ.

Finally, our results have shown evidence that turkey poults in the first month post-hatch can respond to dietary supplementation of phytase doses up to 4,000 FYT/kg of feed in terms of diet utilization. Turkey diets can be formulated with lower Ca and P levels without compromising growth performance and bone mineralization at a young age. The supplementation of turkey poults' diets with high levels of phytase linearly increased P digestibility, P absorption to the bone, MYO provision, nutrient metabolizability, and AME, therefore leading to reduced excretion of dietary protein, Na, Ca, and P.
